# An Absence of Epstein–Barr Virus Reactivation and Associations with Disease Activity in People with Multiple Sclerosis Undergoing Therapeutic Hookworm Vaccination

**DOI:** 10.3390/vaccines8030487

**Published:** 2020-08-28

**Authors:** Peter A. C. Maple, Bruno Gran, Radu Tanasescu, David I. Pritchard, Cris S. Constantinescu

**Affiliations:** 1 Clinical Neurology Research Group, Division of Clinical Neuroscience, University of Nottingham School of Medicine; Queen’s Medical Centre, Nottingham NG7 2UH, UK; Bruno.Gran@nuh.nhs.uk (B.G.); radu.tanasescu@nuh.nhs.uk (R.T.); cris.constantinescu@nottingham.ac.uk (C.S.C.); 2 Department of Neurology, Nottingham University Hospitals NHS Trust; Queen’s Medical Centre, Nottingham NG7 2UH, UK; 3 Department of Neurosciences, University of Medicine and Pharmacy Carol Davila, 021172 Bucharest, Romania; 4 Department of Neurology, Colentina Hospital, 021172 Bucharest, Romania; 5 Immune Regulation Research Group (D.P.), University of Nottingham, Nottingham NG7 2UH, UK; dandt.pritchard@btinternet.com

**Keywords:** multiple sclerosis, Epstein–Barr virus, therapeutic hookworm vaccination, disease activity

## Abstract

**Background:** Epstein–Barr virus (EBV) infection is strongly associated with multiple sclerosis (MS). Helminth infection can downregulate antiviral immune responses, potentially protecting against MS, but with a theoretical risk for reactivating latent EBV infection. **Objective:** To investigate parameters of EBV infection and their relationship with disease activity in people with MS (PwMS) therapeutically vaccinated with *Necator americanus* (hookworm). **Methods:** Sequential serum samples from 51 PwMS; 26 therapeutically infected (25 larvae) with *N. americanus* and 25 controls were tested for EBV virus capsid antigen (VCA) IgG and IgM, EBV nuclear antigen-1 (EBNA-1) IgG, and EBV early antigen (EA) IgG. Disease activity was assessed by periodic MRI. Significance was set at *p* < 0.05. **Results:** All PwMS were EBV VCA IgG and EBNA-1 IgG positive, and 35.2% were EBV EA IgG positive. EBV antibody levels were generally stable, and EBV reactivation in PwMS was not demonstrated by significant increases in IgG titre over 12 months. Disease activity was most frequent in PwMS possessing high levels of EBV VCA IgG (>600 units/mL) or EBNA-1 IgG (>150 units/mL); however, there was no association with hookworm treatment. **Interpretation:** Therapeutic hookworm vaccination was not associated with EBV reactivation. Multiple sclerosis disease activity was associated with high levels of EBV VCA IgG or EBNA-1 IgG.

## 1. Introduction

The hookworms *Necator americanus* and *Ancylostoma duodenale* are nematode roundworms which belong to a larger group of helminth multicellular worms that also include cestode tapeworms and trematode flukes. In hookworm infection, free-living larvae directly penetrate the skin and in the case of *N. americanus*, infecting larvae migrate via the lungs, before attachment and maturation in the gastrointestinal tract. Hookworm infestation is prevalent in countries of Asia, South America and sub-Saharan Africa and is associated with poverty [1,2]. Infection is usually asymptomatic; however, heavy worm loads can trigger intestinal blood loss, leading to iron-deficiency anaemia [3]. Immunologically, helminth infection promotes an anti-inflammatory response by promotion of Th2 immune pathways, stimulation of T and B regulatory cell population activities and the suppression of Th1/Th17 effector responses [4].

An inverse correlation between the distribution of hookworm infestation and the prevalence of allergic and autoimmune diseases has been observed [5]. In the case of multiple sclerosis (MS) [6], an inflammatory demyelinating disease of the central nervous system (CNS), a threshold of helminth prevalence in excess of 10% is associated with a steep decline in the prevalence of disease [7]. Animal models and human studies have both provided evidence supporting an immunomodulatory effect of helminth infection on several autoimmune conditions including the relapsing and remitting form of MS [8]. In view of these associations, there has been considerable interest in the application of helminth therapy for the treatment of multiple sclerosis and several small-scale studies have been reported [8,9].

The risk of adverse virological consequences following suppression of the antiviral Th1 response in hookworm-treated MS patients remains to be established. In animal studies, it has been reported that helminth infection can inhibit antiviral immunity via a pathway of innate immunomodulation [10] and that latent murine gammaherpesvirus infection can be reactivated [11]. Epstein–Barr virus (*Human gammaherpesvirus 4*-EBV) belongs to the family *Herpesviridae*, subfamily *Gammaherpesvirinae* and, following primary infection, establishes latency in human B lymphocytes [12]. Latency is a phenomenon characteristic of all human herpesviruses in which the virus genome persists within selected cells with limited gene expression in a non-replicative state virtually hidden from the host immune response [13]. Under certain conditions, the virus can switch (reactivate) to a replicative/lytic state, revealing itself to the host immune response. The expression of specific virus proteins is associated with EBV latency (e.g., Epstein–Barr nuclear antigen-EBNA-1) or reactivation/active infection (early antigen-EA and/or virus capsid antigen-VCA). Detection of antibodies to these proteins is used to stage EBV infection as recent/active, or past, or reactivation [14]. Infection with Epstein–Barr virus (EBV) is linked to the development of MS [15] and there have been reports of EBV reactivation with disease activity [16,17]. High levels of EBV antibody markers (EBV VCA IgG and EBNA-1 IgG) have also been reported to be associated with increased disease activity in cases of clinically isolated syndrome (CIS) [18] and in PwMS [19].

The Nottingham Worms for Immune Regulation in Multiple Sclerosis (WIRMS) study [20] commenced on 2012 and was a randomized, double-blinded, placebo-controlled clinical trial in which 35 patients were infected with 25 larvae of *Necator americanus* and 36 were given placebo. Samples collected from this study afforded the opportunity to measure selected EBV antibody levels over a period of 12 months, enabling the impact of hookworm therapy on EBV reactivation and other parameters to be assessed. The results of these investigations are described in this study.

## 2. Methods

### 2.1. Study Design and Participants

Serum samples from people with multiple sclerosis (PwMS) were collected over 12 months as part of the WIRMS (Worms for Immune Regulation in MS) trial (NCT 01470521), which was conducted at the Department of Clinical Neurology, University of Nottingham, Nottingham, UK. Appropriate ethical approvals (National Research Ethics Service Committee East Midlands; reference 11/EM/0140) were obtained and all patients provided written informed consent to participate in this study. In this study, which commenced during 2012, 35 PwMS were treated with the hookworm *N. americanus* (25 larvae applied cutaneously per patient) and 36 PwMS received placebo (water). PwMS meeting the McDonald criteria [21] for relapsing and remitting multiple sclerosis were included together with secondary multiple sclerosis patients with superimposed relapse, subject to specific clinical criteria. Exclusion criteria (for details, see supplementary section Tanasescu et al. [20]) included prior or present evidence of parasitic infection and prior treatment with anti-helminthic drugs in the preceding six years. PwMS entered into the trial had not received immunosuppressive drugs 12 weeks prior to enrolment, were immunocompetent, and had not been treated with interferon or glatiramer acetate within 8 weeks of enrolment. *N. americanus* infection was confirmed using real-time polymerase chain reaction and amplicon sequencing [20]. Adequate serum samples were available from 51 study participants: 26 treated with hookworm and 25 given placebo. Multiple aliquots of the serum samples were stored at minus 80 °C following collection.

Multiple sclerosis disease activity (DA) was assessed by the analysis of brain magnetic resonance imaging (MRI) scans performed monthly between months 3 and 9 and again at month 12. Disease activity (DA) was defined as the appearance of new or enlarging lesions on T2 FLAIR imaging or evidence of Gadolinium-DPTA-enhancing T1 lesions.

### 2.2. Detection of EBV Antibodies

Epstein–Barr virus capsid antigen IgG (EBV VCA IgG), EBV capsid antigen IgM (EBV VCA IgM), EBV nuclear antigen-1 IgG (EBNA-1 IgG), and EBV early antigen IgG (EBV EA IgG) antibodies were detected and quantified using SERION ELISA *classic* (Institut Virion\Serion GmbH, Würzburg, Germany) kits. The manufacturer’s instructions were followed.

The cut-offs applied were as specified by the manufacturer and were as follows: EBV VCA IgG (<10 U/mL = negative; >15 U/mL = positive), EBV VCA IgM (<9 U/mL = negative; >13 U/mL = positive), EBV EA IgG (<10 U/mL = negative; >15 U/mL = positive), and EBNA-1 IgG (<2.5 U/mL = negative; >3 U/mL = positive). Test results falling between the negative and positive cut-offs were graded equivocal.

### 2.3. Statistical Methods

Basic data manipulations, for example, calculation of geometric means and standard deviations together with graphical presentations, were performed using Excel 2016 and GraphPad Prism (version 8). The level of significance for statistical analyses was set at *p* < 0.05. Associations of categorical data (e.g., seroprevalence) were tested using Fisher’s exact test because of the small sample sizes available. Normality was estimated using Shapiro–Wilk and Kolmogorov–Smirnov tests. The Mann–Whitney U test was used to evaluate associations of independent samples where normality could not be assumed.

## 3. Results

### 3.1. Demographics of PwMS Groups

There were 26 PwMS in the hookworm-treated group, comprising 21 females and 5 males, and there were 25 PwMS in the placebo-controlled group, comprising 19 females and 6 males. The mean age of PwMS in the hookworm-treated group was 44.7 years and the mean age of the PwMS in the placebo-controlled group was 45.2 years. Clinical characteristics of the PwMS recruited to the trial are shown in Table 1. Apart from three South Asians, all trial participants were Caucasians. There were no serious clinical adverse events directly attributable to EBV during the trial. Infection with *N. americanus* was confirmed using molecular methods in 23 of 35 PwMS who underwent therapeutic infection [20].

### 3.2. EBV VCA IgM Seroprevalence in the Hookworm- and Placebo-Treated Groups

Only one PwMS in the hookworm-treated group was EBV VCA IgM positive and only one PwMS in the placebo-controlled group was EBV VCA IgM positive. Both patients were IgM seropositive throughout the 12 month study period: in the hookworm-treated PwMS, IgM levels ranged over time between 40 and 70 U/mL (mean 52.7 U/mL); in the placebo control, PwMS IgM levels ranged between 25 and 30 U/mL (mean 27 U/mL). Both of the above PwMS were seropositive for EA IgG, EBNA-1 IgG and VCA IgG markers.

### 3.3. EBV VCA IgG and EBNA-1 IgG Seroprevalence and Levels in the Hookworm- and Placebo-Treated Groups

**Figure 1:**
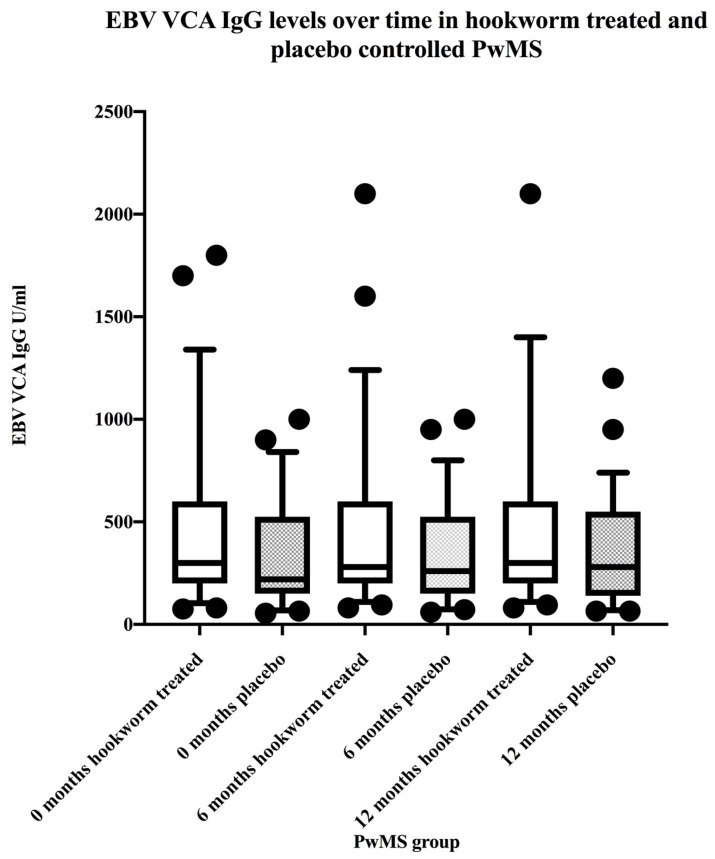
Data represent stability over 12 months of EBV VCA IgG levels in hookworm-treated and placebo-controlled PwMS. Box and whiskers plots comparing median and 10th and 90th percentile range with outliers of EBV VCA IgG levels in hookworm-treated (clear) versus placebo-controlled (shaded) PwMS before, during, and after treatments.

**Figure 2:**
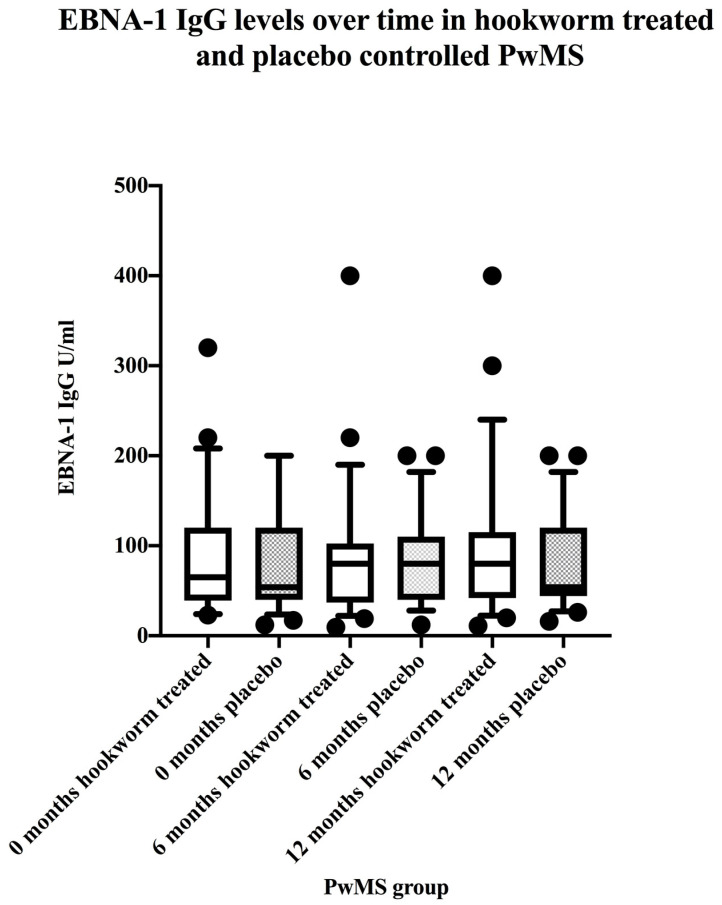
Data represent stability over 12 months of EBNA-1 IgG levels in hookworm-treated and placebo-controlled PwMS. Box and whiskers plots comparing median and 10th and 90th percentile range with outliers of EBNA-1 IgG levels in hookworm-treated (clear) versus placebo-controlled (shaded) PwMS before, during, and after treatments.

All PwMS were seropositive for EBV VCA IgG and VCA IgG levels were stable over the 12 month study period (Figure 1). All PwMS were seropositive for EBNA-1 IgG and EBNA-1 IgG levels were stable over the 12 month study period (Figure 2). There were no differences between EBV VCA IgG levels in hookworm-treated PwMS and placebo controls before, during, or after treatment (Table 2). Similarly, there were no differences between EBNA-1 IgG levels in hookworm-treated PwMS and placebo controls before, during, or after treatment (Table 2).

### 3.4. EBV EA IgG Seroprevalence and EBV Reactivation in the Hookworm- and Placebo-Treated Groups

In the hookworm-treated PwMS, 14 PwMS were EA IgG negative, 4 were equivocal, and 8 (30.7%) were EA IgG positive (range 16 U/mL–65 U/mL). In the placebo-controlled group, 12 PwMS were EA IgG negative, 3 were equivocal, and 10 (40.0%) were EA IgG positive (range 16 U/mL–100 U/mL). Generally, EA IgG levels were stable over time, but some patients showed more variation than others. The difference (treating equivocals as seronegatives) in the EBV EA IgG seropositivity between the two groups was not significant (Fisher exact test statistic value = 0.565). The detection of EBV EA IgG in the presence of EBV VCA IgG and EBNA-1 IgG is potentially consistent with virus reactivation so there is a theoretical possibility that approximately 30–40% of PwMS tested showed evidence of EBV reactivation. In all these cases, the pre-treatment serum was EBV EA IgG positive and there was no evidence of a trend of increasing EA IgG levels during the 12 months of monitoring which suggests the existence of processes not consistent with classical reactivation.

### 3.5. Stratification of Levels of EBV VCA IgG and EBNA-1 IgG and Association with Disease Activity in PwMS

**Figure 3:**
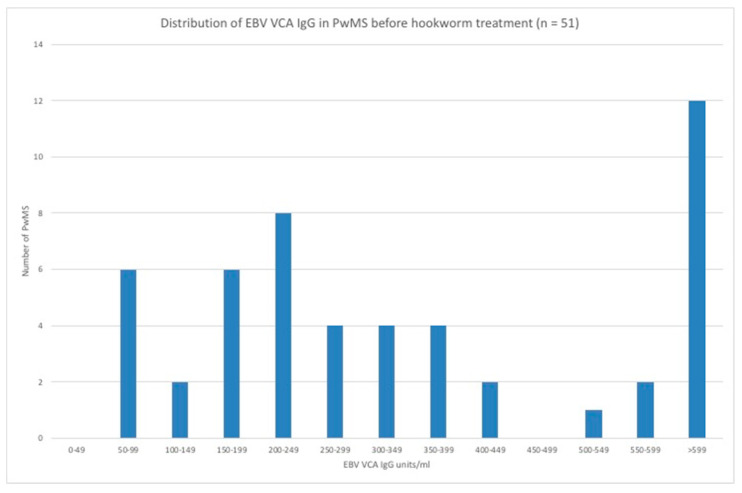
Distribution of EBV VCA IgG levels in PwMS at baseline.

**Figure 4:**
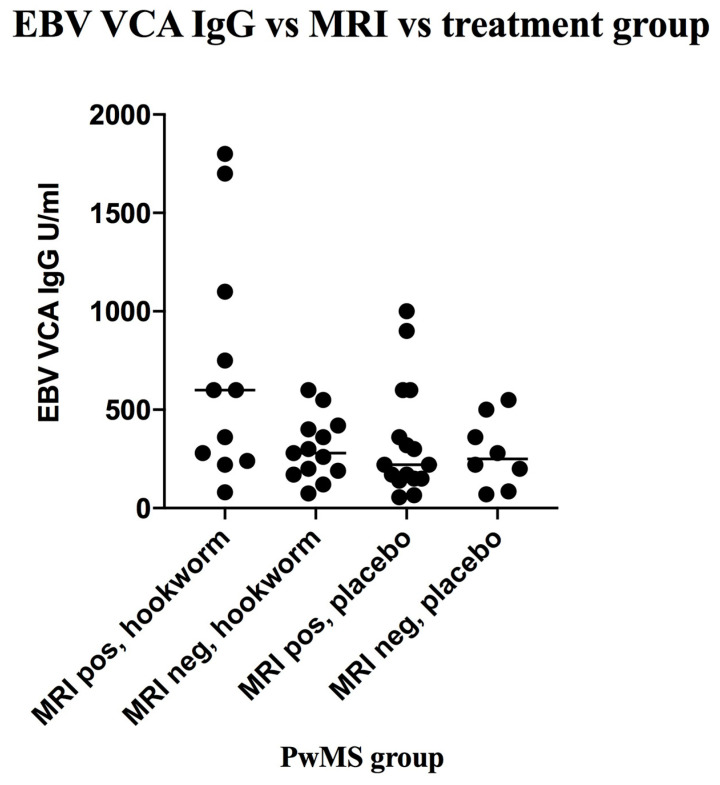
Scatter plot of EBV VCA IgG levels (baseline data, n = 48) in hookworm-treated versus placebo-controlled groups of people with multiple sclerosis (PwMS) differentiated by disease activity. MRI = brain magnetic resonance imaging, and positivity was the development of new or enlarging lesions (see methods).

The distribution of EBV VCA IgG levels in PwMS is shown in Figure 3. MRI data was available for 48 PwMS. There were 27 (56.2%) PwMS who showed new disease activity (DA)—11 had been therapeutically infected with hookworm and 16 had received placebo (Figure 4). The geometric mean EBV VCA IgG level of the new DA group was 325 units/mL (95% CI: 224–470 units/mL) and that of the no new DA group was 247 units/mL (95% CI: 184–332 units/mL). Stratification of PwMS (n = 48) by their EBV VCA IgG levels showed that there were 11 (22.9%) PwMS with EBV VCA IgG levels greater than or equal to 600 units/mL, comprising 10 (90.9%) PwMS who showed new DA and 1 (9.1%) PwMS who showed no new DA (Figure 4). The group of PwMS with high EBV VCA IgG levels (≥600 units/mL) was deemed high risk for new DA and differed significantly from the lower-risk (<600 units/mL) PwMS. In those PwMS who were hookworm treated (Figure 4) and showed new DA, 6/11 (54.5%) were in the EBV VCA IgG high-risk group (levels ≥600 units/mL). This compared with 4/16 (25.0%) of EBV high-risk group PwMS who received placebo. There was no significant difference in new DA of EBV VCA IgG high-risk PwMS who received hookworm treatment and those who received placebo.

**Figure 5:**
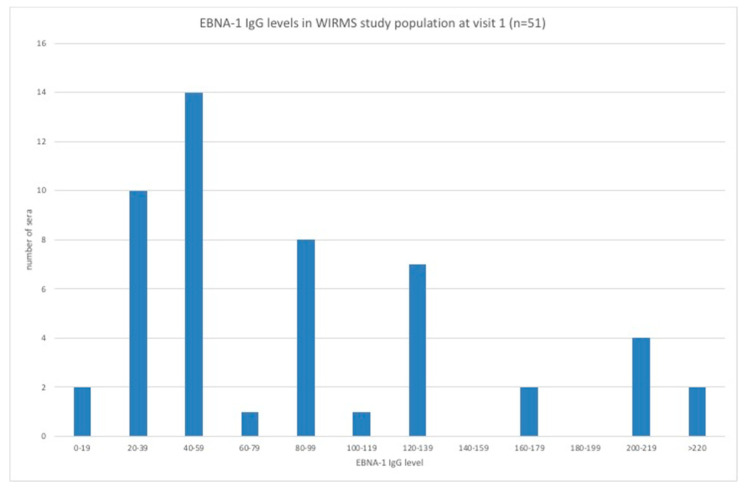
Distribution of EBNA-1 IgG levels in PwMS at baseline.

**Figure 6:**
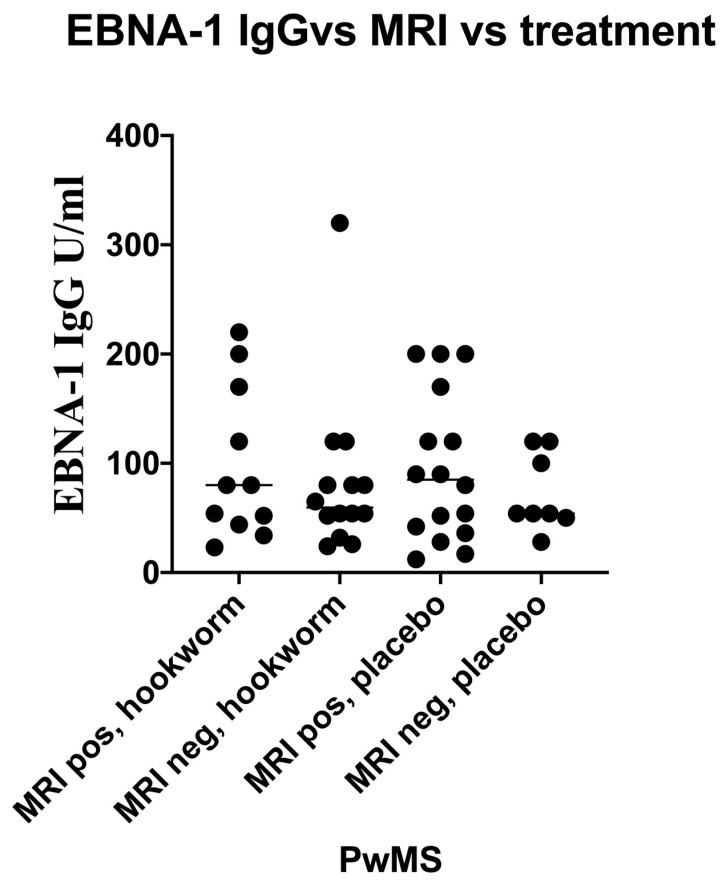
Scatter plot of EBNA-1 IgG levels (baseline data, n=49)) in hookworm-treated versus placebo-controlled groups of people with multiple sclerosis (PwMS) differentiated by disease activity. MRI = brain magnetic resonance imaging, and positivity was the development of new or enlarging lesions (see methods).

The distribution of EBNA-1 IgG levels in PwMS is shown in Figure 5. MRI data was available for 49 PwMS. There were 27 (55.1%) PwMS who showed new disease activity (DA) and 22 (44.9%) PwMS who showed no new DA (Figure 6). The geometric mean EBNA-1 IgG level of the new DA group was 72.5 units/mL (95% CI: 52.5–100.1 units/mL) and that of the no new DA group was 65.1 units/mL (95% CI: 49.7–85.2 units/mL). Stratification of PwMS by their EBNA-1 IgG levels showed that there were 8 (16.3%) PwMS with EBNA-1 IgG levels greater than or equal to 150 units/mL, comprising 7 (87.5%) PwMS who showed new DA and 1 (12.5%) PwMS who showed no new DA (Figure 6). The group of PwMS with high EBNA-1 IgG levels (≥150 units/mL) was deemed high risk for new DA and differed significantly from the lower-risk (<150 units/mL) PwMS. In those PwMS who were hookworm treated (Figure 6) and showed new DA, 3/11 (27.2%) were in the EBNA-1 IgG high-risk group (levels ≥150 units/mL). This compared with 4/16 (25.0%) of EBV high-risk group PwMS who received placebo. There was no significant difference in new DA of EBV VCA IgG high-risk PwMS who received hookworm treatment and those who received placebo.

## 4. Discussion

The Nottingham WIRMS study (NCT 01470521) is the largest clinical trial of hookworm therapy for MS to be conducted in adults and the first to be placebo controlled. The rationale for this study was that natural hookworm infection promoted an anti-inflammatory Th2 and regulatory T cell immune phenotype, which would be beneficial for PwMS who typically show regulatory B and T cell dysfunction [22,23] leading to excessive inflammatory activation and a predominant Th1 immune phenotype. A concern of this form of treatment intervention was that the antiviral Th1 response may be excessively compromised, resulting in reduced control of virus infections. Laboratory studies using murine models of infection [10,11] have raised the possibility of an increased potential for EBV reactivation. The clinical sequelae of such reactivations may be severe [24] and it is relevant that herpesvirus reactivations be assessed in patients receiving this form of treatment.

Unlike VZV reactivation, where a shingles-type rash is an obvious, although not definitive, sign of renewed virus activity, there is no overt clinical sign of EBV reactivation; therefore, appropriate laboratory investigations are essential. In immunocompetent individuals, detection of relevant antibody markers by serological tests is the widely adopted approach [25] for staging EBV infection as “acute” or “past”. Virus replication (lytic phase) either during primary infection or reactivation is evidenced by the detection of virus capsid antigen (VCA) IgM and IgG and early (EA) IgG. Virus latency (past infection) is evidenced by the detection of specific IgG to nuclear antigens, typically EBNA-1. EBV reactivation can be evidenced directly by the detection of virus DNA using quantitative EBV viral load assays [26]; however, a limitation of viral load assays is their variability and lack of specificity in differentiating EBV reactivation from periodic virus shedding and for this reason they were not used in our study. In our study, no PwMS seroconverted to EBV VCA IgM or were EBV EA IgG seropositive during the study period. Persistent EBV EA antibody levels were observed in the hookworm-treated group, in which 30.7% were EA IgG seropositive, and in the placebo-controlled group, in which 40.0% were EA IgG seropositive. The difference in the EBV EA IgG seropositivity between the two groups was not significant. Although traditionally considered as a marker for EBV reactivation, the significance of our finding of EA IgG seropositivity is unclear, particularly, as individual EA IgG levels remained constant throughout the 12 months of monitoring. Furthermore, a background EA IgG seropositivity of approximately 10–20% has been reported in several studies [27,28] and on testing a panel of 124 control sera [29] collected from adults living in the Nottingham area, we observed a background EBV EA seropositivity of 21.5% (data not reported). These findings merit further investigation.

An interesting observation derived from our study is the existence of subpopulations of PwMS with high levels of either EBV VCA IgG (≥600 units/mL) or EBNA-1 IgG (≥150 units/mL) who appeared at higher risk of disease activity. An association between levels of EBV VCA IgG or EBNA-1 IgG and disease activity has been observed by others. For example, Horakova and colleagues [30] reported in a study of clinically isolated syndrome patients that the cumulative number of contrast-enhancing lesions and T2 lesions during a two-year period was greater for individuals comprising the highest quartile of EBV VCA IgG levels. In another study, Jakimovski and colleagues [31] showed high EBNA-1 IgG levels to be associated with focal destructive lesion pathology in RRMS patients.

## 5. Conclusions

In conclusion, there was no evidence of EBV reactivation in PwMS following therapeutic infection with small numbers (25 larvae) of hookworm for a period of nine months. Previous studies [32,33,34] have shown the complexity of humoral and cellular immune responses following hookworm/helminth infections and a highly significant negative correlation between total IgE levels and hookworm weight and fecundity has been documented [35]. Our findings relate only to the particular conditions of our study in which a low parasite burden of infection was established [36] and the data presented add to our knowledge of the safety profile [37,38] of therapeutic hookworm infection. Finally, it appears that subsets of PwMS with high levels of EBV antibodies are at greater risk of disease activity and this observation requires further investigation as it raises the possibility of targeted anti-EBV interventions to reduce disease activity.

## Figures and Tables

**Table 1:** Demographics and baseline clinical features of hookworm-treated (*n* = 26) and placebo-controlled (*n* = 25) PwMS.

Patient Characteristics	Hookworm	Placebo
Demographic characteristics		
Age		
(mean age, range)	44.8 (32–60)	49.3 (29–59)
Sex (no. of subjects, %)		
Female	21 (81%)	19 (76%)
Male	5 (19%)	6 (24%)
Clinical characteristics		
Type of relapsing MS		
(no. of subjects, %)	21 (81%)	21 (84%)
RR		
SP with superimposed relapses	5 (19%)	4 (16%)
Time from first symptoms of MS (mean years, SD)	11 (8.3)	9.2 (9.3)
Mean EDSS score (range)	3 (1.5–5.5)	2.6 (1.5–4)

**Table 2:** EBV VCA and EBNA-1 geometric mean IgG levels in hookworm-treated (*n* = 26) and placebo-controlled (*n* = 25) PwMS before, during, and three months after treatment.

EBV Antibody and Treatment Group	Before Treatment (0 months)	During Treatment (6 months)	Post Treatment (12 months)
EBV VCA IgG	340.2 units/mL	347.2 units/mL	351.1 units/mL
Hookworm treated	95% CI: [242–476.3]	95% CI: [248–486]	95% CI: [250–493]
EBV VCA IgG	251.4 units/mL	257.0 units/mL	254.8 units/mL
Placebo	95% CI: [179–352]	95% CI: [185–355]	95% CI: [183–354]
EBNA-1 IgG	67.8 units/mL	64.7 units/mL	65.7 units/mL
Hookworm treated	95% CI: [50.2–91.6]	95% CI: [45.9–91.3]	95% CI: [46.6–92.6]
EBNA-1 IgG	66.4 units/mL	65.3 units/mL	67.0 units/mL
Placebo	95% CI: [48.6–90.9]	95% CI: [49–86.8]	95% CI: [50.4–89]
